# Predicting amyloid status in mild cognitive impairment: the role of semantic intrusions combined with plasma biomarkers

**DOI:** 10.3389/fnagi.2025.1624513

**Published:** 2025-06-25

**Authors:** Yao Lu, Liang Cui, Lin Huang, Fang Xie, Qi-Hao Guo

**Affiliations:** ^1^Department of Gerontology, Shanghai Sixth People's Hospital, Shanghai Jiao Tong University School of Medicine, Shanghai, China; ^2^Department of Nuclear Medicine & PET Center, Huashan Hospital, Fudan University, Shanghai, China

**Keywords:** mild cognitive impairment, semantic intrusions, BVLT, p-tau217, GFAP, amyloid

## Abstract

**Background:**

The efficacy of traditional semantic intrusion measurements in identifying amyloid deposition in mild cognitive impairment (MCI) patients remains suboptimal. It is anticipated that integrating innovative cognitive assessments with blood biomarker analyses will enhance the effectiveness of screening for Alzheimer’s disease (AD).

**Methods:**

The research included 204 participants from the Chinese Preclinical Alzheimer’s Disease Study cohort, assessed between March 2019 and February 2023. The Bi-list Verbal Learning Test (BVLT) was utilized to measure semantic intrusions, while amyloid burden was quantified using neuroimaging with 18F-florbetapir PET/CT scans. Additionally, the study analyzed Apolipoprotein E loci and plasma biomarkers, including Aβ42, Aβ40, Tau, p-tau181, p-tau217, Nfl, and GFAP.

**Results:**

The study revealed that semantic intrusion errors on the BVLT are highly predictive of amyloid deposition in MCI participants. Binary logistic regression analysis confirmed that semantic intrusion errors on the Bi-list Verbal Learning Test, along with p-tau217 levels and GFAP levels, can effectively predict amyloid positive MCI. Correlation analysis further established a positive association between p-tau217, GFAP, and semantic intrusion errors among patients with A+ MCI. The combined predictors (p-tau217, GFAP, semantic intrusion errors) demonstrated outstanding performance in ROC analysis, achieving an AUC of 0.964, with a sensitivity of 92.7% and a specificity of 85.7%.

**Conclusion:**

The study suggests that semantic intrusion errors from the BVLT, along with plasma biomarkers p-tau217 and GFAP, may serve as sensitive indicators for AD-related MCI. Combining these biomarkers with semantic intrusion errors offers a strong predictive model for assessing amyloid status in MCI patients.

## Introduction

According to the 2024 research framework established by the Alzheimer’s Association (AA) (Jack et al., 2024), individuals with brain Aβ deposits, identified through amyloid-PET or cerebrospinal fluid, are considered in the early stages of Alzheimer’s disease (AD), including those with normal cognition or mild cognitive impairment (MCI). The current trajectory of Alzheimer’s disease treatment development is predominantly guided by the amyloid hypothesis, concentrating on individuals who are amyloid-positive and in the prodromal or initial stages of the disease. Research has confirmed that in the Chinese population, the overall amyloid positivity rate among individuals with MCI is 44.5% (He et al., 2024). Early detection of amyloid positivity can be crucial for managing the disease and planning future care. However, there remains a deficiency in cognitive assessment tools that possess the requisite sensitivity and specificity to effectively monitor changes associated with this biomarker (Loewenstein et al., 2018b).

Conventional cognitive assessment techniques pose substantial challenges for the early detection of AD, as they frequently lack the sensitivity required to detect the initial cognitive deficits indicative of the condition. The Loewenstein Acevedo Scales of Semantic Interference and Learning (LASSI-L) has emerged as a promising instrument, exhibiting enhanced sensitivity to early cognitive impairments when compared to traditional memory assessment tools, which primarily focus on impaired learning or accelerated forgetting rates (Loewenstein et al., 2018a,b; Matias-Guiu et al., 2018). Loewenstein et al. (2018a) discovered that semantic intrusion errors, which are indicative of proactive semantic interference (PSI) and failure to recover form PSI (frPSI), could effectively distinguish between amyloid-positive aMCI patients and amyloid-negative aMCI patients. PSI occurs when prior learning of similar items hinders the acquisition of new information. The frPSI indicates that these interference effects continue to impact memory even after multiple learning attempts (Loewenstein et al., 2017a,b). In the LASSI-L assessment, immediate recall is evaluated following the presentation of the word list. However, it remains uncertain whether PSI and frPSI during delayed recall exhibit greater sensitivity in detecting amyloid-positive MCI. The Bi-list Verbal Learning Test (BVLT) represents a Chinese adaptation of the LASSI-L, developed by the authors in accordance with its design principles. In consideration of Chinese cultural context, modifications were made to the items requiring memorization; for instance, musical instrument names such as “guitar,” “three-stringed instrument,” and “bell” were replaced with more easily recognizable animal names. Furthermore, during the preliminary validation of the LASSI-L’s applicability, it was observed that the original set of 15 words presented a considerable level of difficulty, prompting a reduction to 12 words. Given that delayed recall serves as a sensitive indicator of cognitive impairment, the BVLT integrates the semantic interference detection alongside the assessment of delayed recall, which enhances the assessment’s validity. To the best of our knowledge, no prior studies have investigated the clinical utility of PSI and frPSI during delayed recall in relation to Alzheimer’s disease biomarkers.

Blood biomarkers, including P-tau217, P-tau181, and P-tau231, have been integrated into the AD diagnostic criteria established by the AA. The combination of sophisticated cognitive assessments with blood biomarker analysis is expected to improve the effectiveness of AD screening (Aye et al., 2025; Weiner et al., 2023)^.^ However, there remains a significant lack of comparable research in this domain. This study aimed to evaluate and validate the efficacy of the novel BVLT alongside various blood biomarkers, in diagnosing amyloid-positive MCI within the Chinese population.

## Method

### Participants

This study enrolled participants from the Chinese Preclinical Alzheimer’s Disease Study (C-PAS) cohort (Cui et al., 2023), a longitudinal study led by Qi-Hao Guo that began in 2019 at Shanghai Sixth People’s Hospital. The study aims to identify mechanisms and detect preclinical Alzheimer’s disease in Chinese population. Participants were recruited from a memory clinic or the community. The C-PAS has the following inclusion criteria: native Chinese speaker; aged over 50 years; adequate visual and auditory acuity for neuropsychological testing; no severe illness precluding enrollment. They underwent assessments including medical history, neurological exams, neuropsychological testing [Auditory Verbal Learning Test (Zhao et al., 2015), Montreal Cognitive Assessment-B (Huang et al., 2018), Addenbrooke’s Cognitive Examination III (Pan et al., 2022b), Functional Assessment Questionnaire (Pfeffer et al., 1982), Activities of Daily Living (Chen et al., 1995)], neuroimaging, and lab tests. Baseline data of 204 participants from the C-PAS were included in the current study between September 2019 and February 2023. Amyloid- PET/CT, Apolipoprotein E (APOE) genotyping, plasma biomarkers, and a battery of standardized neuropsychological assessments were completed for all participants. Individuals were then classified into three groups based on their cognitive status and PET/CT results.

The major grouping was based on the stratification of cognitive functions and amyloid deposition status. (1) Amyloid negative normal cognition (A− NC, *n* = 69): normal subjective sensory cognition. Amyloid negativity was confirmed by PET/CT. (2) MCI: individuals were classified as having MCI based on an actuarial neuropsychological method (Bondi et al., 2014). According to this method, a diagnosis of MCI was given if the participant met the following criteria: ① Impaired scores (defined as >1 standard deviation below the age-corrected normative mean) on two indexes within the same cognitive domain (memory, language or executive function); ② Impaired scores (defined as >1 SD below the age-corrected normative mean) in each of the three cognitive domains. Participants were categorized into two groups based on amyloid deposition status: amyloid-positive MCI group (A+ MCI, *n* = 70) and amyloid-negative MCI group (A− MCI, *n* = 65).

### Bi-list verbal learning test (BVLT)

The procedure for administering the test is as follows: At first, the examiner presents a list of 12 common words, categorized into fruits, animals, and countries (4 words from each category) known as List A. Once all 12 words have been read by the examiner, the participant is asked to recall them. After a free recall (Free Recall, N1), the participant is given semantic cues (e.g., “Now I want you to tell me all the words from the list that are fruits”) with 20 s allocated for each category (Cued Recall A1, N2). List A is then presented again using the same method, followed by another free recall (N3). Subsequently, a semantically related list (List B) with 12 common words from the same categories is introduced using the same procedure, followed by free recall (N4) and cued recall (Cued recall B1, N5). Afterward, List B is presented once more, concluding with a second cued recall attempt (Cued recall B2, N6). Then there is an interval period (The participant is asked to calculate 91 minus 3, then subtract 3 again, and keep subtracting 3 for 60 s). The participant is then asked to freely recall the words from List A (N7), which is followed by a category-cued recall trial (Short-delay cued recall A3, N8). Another interval period (subtracting 3 from 92 for 60 s) is followed by the free recall of List B (N9). Then a delayed recall test for List B given semantic cues is conducted (Short delay cued recall B3, N10). Finally the recognition test is conducted (N11). The test records correctly remembered words, intrusions from the other list, and unrelated intrusions. Participants may take about 10 to 15 min to complete the test.

### Imaging data, genetics and plasma biomarkers

All participants underwent 18F-florbetapir PET/CT scans (Biography 64 PET/CT, Siemens, Erlangen, Germany) at the PET Center, Huashan Hospital, Fudan University. The scans were performed 50 min after intravenous administration of ~7.4 MBq/kg of F18-florbetapir. The assessment to classify the 18F-florbetapir PET scans as positive or negative was conducted using the visual evaluation method, following the foundational guidelines provided by the Society of Nuclear Medicine and Molecular Imaging (SNMMI) (Minoshima et al., 2016) and the International Nuclear Medicine consensus on the clinical application of Amyloid PET in Alzheimer’s Disease (Tian et al., 2023). 3.0 T MRI scans were performed (Prisma 3.0 T, Siemens, Erlangen, Germany) at Shanghai Sixth People’s Hospital, Shanghai Jiao Tong University School of Medicine.

APOE genotyping was also examined using PCR techniques, and participants were classified as APOE ε4 carriers if they had at least one ε4 allele. Concentrations of plasma Aβ42, Aβ40, t-tau, p-tau181, p-tau217, glial fibrillary acidic protein (GFAP) and neurofilament light chain (Nfl) were measured via Single Molecular Array (SiMoA) assays (Pan et al., 2022a).

### Data analysis

Given the presence of three diagnostic groups with interval-level data, a series of one-way analyses of variance (ANOVA) were conducted. When a statistically significant *F*-value was obtained (*p* < 0.05), post-hoc comparisons were performed using the Bonferroni test, with significance set at *p* ≤ 0.05. For categorical variables, chi-square analyses were used, with significance also set at *p* < 0.05. Further analysis was conducted using binary logistic regression models to explore the associations between plasma biomarkers (Aβ1-42/Aβ1-40 ratio, p-tau217, p-tau181, GFAP, and NfL) and SI errors—considered as independent variables (exposures)—and A + MCI as the dependent variable. The models were adjusted for potential confounding factors, including age, sex, education, and APOE status. Using Spearman correlation analysis to explore the correlation between blood biomarker levels, SI errors, and Aβ status. Receiver operator characteristic (ROC) curves were calculated for the SI errors and plasma biomarkers to determine their ability to classify A+ MCI from A− MCI. Statistical analysis was performed using SPSS version 22.0. Figures were produced using GraphPad Prism version 9.

## Results

### Demographics, biomarkers and neuropsychological tests of participants in Alzheimer’s continuum

Table 1 presents demographic and clinical characteristics of the total cohort, comprising 204 participants with amyloid-PET scans. The median age of the overall sample was 67 years, ranging from 52 years to 84 years. No significant differences were observed among the subgroups of amyloid positive MCI (A+ MCI), amyloid negative MCI (A− MCI), and amyloid negative normal controls (A− NC) with respect to age, gender or educational levels. The A+MCI group and A− MCI group had a significantly higher prevalence of APOE ε4 carriers compared to the A− MCI group and normal control group. In terms of plasma biomarkers, no differences were detected among the groups for t-tau, Aβ1-42, Aβ1-40, or the Aβ1-42/Aβ1-40 ratio. Significant differences were observed among the groups in terms of p-tau181, p-tau217, NfL and GFAP levels, with elevated concentrations in the A+ MCI group compared to the A− NC group. Additionally, significant differences in p-tau217, NfL and GFAP levels were observed between the A+MCI group and the A− MCI group.

**Table 1:** Demographics, biomarkers and neuropsychological tests of participants in Alzheimer’s continuum.

Characteristic	Amy− NC (*n* = 69)	Amy− MCI (*n* = 70)	Amy+ MCI (*n* = 65)	*p* value
Age (years)	63.93 (6.21)	64.09 (8.69)	64.11 (5.91)	0.633
Gender (female, %)	34 (49.28)	35 (50.00)	34 (52.31)	0.049
Education (years)	12.88 (3.72)	12.06 (2.16)	11.96 (3.08)	0.057
ApoE (e4 carrier/total, %)	12 (17.39)	14 (20.00)	19 (29.15)^†,‡^	**<0.001**
T-tau (pg/ml)	2.83 (1.24)	2.69 (1.43)	2.83 (1.00)	0.474
p-tau181 (pg/mL)	1.31 (0.84)	1.70 (1.21)	1.92 (0.94)^†^	**0.045**
p-tau217 (pg/ml)	0.31 (0.08)	0.37 (0.20)^‡^	0.54 (0.35)^†,‡^	**<0.001**
Aβ1-40 (pg/mL)	201.38 (67.59)	211.40 (86.53)	207.71 (80.27)	0.450
Aβ1-42 (pg/mL)	10.19 (3.61)	10.82 (4.43)	10.43 (4.36)	0.734
Aβ_1-42_/Aβ_1-40_ ratio	0.058 (0.023)	0.054 (0.021)	0.052 (0.011)	0.482
NfL (pg/mL)	24.68 (11.81)	25.31 (6.90) ^‡^	28.14 (12.19)^†,‡^	**0.023**
GFAP (pg/mL)	112.98 (41.58)	121.13 (57.76)^‡^	159.26 (68.71)^†,‡^	**<0.001**
Neuropsychology test				
AVLT				
AVLT-N3	8.22 (1.96)	6.62 (2.04)^*^	6.04 (1.82)^†^	**0.002**
AVLT-N4	6.78 (2.46)	3.74 (2.73)^*^	3.58 (2.58)^†^	**<0.001**
AVLT-N5	6.56 (3.09)	3.00 (2.55)^*^	2.78 (2.73)^†^	**<0.001**
AVLT-N6	6.11 (3.90)	4.08 (4.17)	3.09 (2.87)^†^	**0.038**
AVLT-N7	22.22 (1.35)	19.65 (4.30)^*^	19.05 (3.01)^†^	**0.023**
MoCA-B	25.57 (2.31)	22.26 (3.62)^*^	21.81 (4.08)^†^	**<0.001**
ACEIII	82.76 (6.38)	78.83 (8.67)	74.00 (9.67)^†^	**0.008**
ADL	20.10 (0.48)	20.18 (0.29)	20.70 (1.48)	0.090
FAQ	0.82 (1.20)	1.13 (3.09)	1.21 (3.64)^†^	**0.048**

Amy+, amyloid positive; Amy−, amyloid negative; NC, normal cognition; MCI, mild cognitive impairment; AVLT, Auditory verbal learning test, MoCA-B, Montreal Cognitive Assessment Basic; ACEIII, Addenbrooke’s Cognitive Examination III; FAQ, Functional Activity Questionnaire; ADL, Activity of Daily Living Scale; GFAP, glial fibrillary acidic protein; NfL, neurofilament light chain. AVLT-N3: correct recalls after the third study trial. AVLT-N4: short delay free recall (5 min). AVLT-N5: long delay free recall (20 min). AVLT-N6: long delay cued recall. AVLT-N7: recognition. ^*^A− NC group compared with A− MCI group, corrected *p* < 0.05. ^†^A−NC group compared with A+ MCI group, corrected *p* < 0.05. ^‡^A− MCI group compared with A+ MCI group, corrected *p* < 0.05. ^*,†,‡^Means with different superscripts are statistically significant *p* < 0.05 by the Bonferroni Test. Bold values: *p* < 0.05.

Regarding to neuropsychological assessments, there were notable differences in the AVLT, MoCA-B, ACEIII, FAQ between the A+MCI group and A− NC group. Also, significant differences in AVLT-N3, AVLT-N4, AVLT-N5, AVLT-N7 and MoCA-B were observed between the A− MCI group and the A− NC group.

### Performance of different diagnostic groups on BVLT measures

The Table 2 displays the comparison of different types of semantic intrusion errors between the A+ MCI group, A− MCI group, and A− NC group. Proactive semantic intrusion (PSI), failure to recover from PSI (frPSI), and retroactive interference (RSI) are the different indicators of semantic intrusions. Notably, the BVLT scale has incorporated delayed recall indicators for semantic intrusions (PSI2, PSI3, RSI1, RSI2). Table 2 provides a comparative analysis of semantic intrusion errors among the group of A− NC, A− MCI and A+ MCI. The data reveals that the A+ MCI group exhibits significantly higher SI errors compared to both the A− NC and Amy− MCI groups, particularly in the PSI2, PSI3, and frPSI measurements. The number of correct responses for each item on the BVLT across three groups are displayed in Supplementary Figure 2. The A+MCI group has significantly fewer correct responses on items N2, N3, N5, N6, N7, N9, N10, and N11 compared to the A− NC group. The A− MCI group differs significantly from the A− NC group on items N6, N9, and N10. Additionally, a significant difference exists between the A− MCI and A+ MCI groups on item N6.

**Table 2:** Comparison of different types of semantic interference errors and neuropsychology test between Amy+ MCI−, Amy− MCI−, and Amy− NC.

BVLT characteristic	Amy− NC (*n* = 69)	Amy− MCI (*n* = 70)	Amy+ MCI (*n* = 65)	*p* value
PSI1 (mean, SD)	0.80 (1.05)	1.04 (1.21)	1.28 (1.11)	0.178
PSI2 (mean, SD)	1.47 (1.60)^*,†^	1.41 (1.58)^*,‡^	2.90 (1.66)^†,‡^	**<0.001**
PSI3 (mean, SD)	1.72 (1.72)^*,†^	2.73 (1.99)^*,‡^	3.34 (1.96)^†,‡^	**<0.001**
frPSI (mean, SD)	0.87 (1.01)^*,†^	1.46 (1.31)^*,‡^	2.88 (1.47)^†,‡^	**<0.001**
RSI1 (mean, SD)	2.46 (2.03)	2.79 (1.97)	2.50 (1.83)	0.583
RSI2 (mean, SD)	2.73 (1.99)	3.53 (3.93)	3.85 (2.50)	0.087

PSI1, Number of items from Group B intruded into Group A during the N4 (free recall). PSI2, Number of items from Group A intruded into Group B during the N7 (short delay recall). PSI3, Number of items from Group B intruded into Group A during the N9 (short delay recall). frPSI, Number of items from Group A intruded into Group B during the N6 (cued recall). RSI1, Number of items from Group B intruded into Group A during the N8 (short delay cued recall). RSI2, Number of items from Group A intruded into Group B during the N10 (short delay cued recall). ^*^A− NC group compared with A− MCI group, corrected *p* < 0.05. ^†^A− NC group compared with A+ MCI group, corrected *p* < 0.05. ^‡^A− MCI group compared with A+ MCI group, corrected *p* < 0.05. ^*,†,‡^Means with different superscripts are statistically significant *p* < 0.05 by the Bonferroni Test. BVLT, Bi-list verbal learning test; PSI, proactive semantic interference; frPSI, failure to recover from proactive semantic interference; RSI, retroactive semantic interference. Bold values: *p* < 0.05.

### Associations of plasma biomarkers, sematic intrusions, demographic factors and amyloid status of MCI participants

Table 3 presents the findings from a binary logistic regression analysis aimed at predicting A+ MCI. This analysis considered a range of predictors, encompassing sex, age, years of education, APOE genotype, Aβ1-42/Aβ1-40 ratio, p-tau217 levels, p-tau181 levels, NfL levels, GFAP levels, and semantic intrusion (SI) errors as measured by PSI3, PSI2, and frPSI. The analysis revealed that p-tau217, GFAP, PSI3, and frPSI are significant predictors for A+ MCI. Specifically, elevated levels of p-tau217 (OR = 2.686, 95% CI 1.003–3.735, *p* = 0.014) and GFAP (OR = 1.926, 95% CI 1.036–2.384, *p* = 0.025), along with a greater number of intrusion errors in PSI3 (OR = 1.470, 95% CI 1.002–1.978, *p* = 0.043) and frPSI (OR = 1.627, 95% CI 0.979–2.085, *p* = 0.035), are associated with an increased likelihood of A+ MCI.

**Table 3:** Coefficients from binary logistic regression analysis to predict Amy+ MCI.

Outcome	Predictors	OR	95%CI	*p* value
A+ MCI	Sex	0.942	0.215–4.120	0.937
	Age	0.916	0.785–1.068	0.263
	Education years	0.906	0.738–1.113	0.153
	APOE	0.818	0.155–4.322	0.813
	Aβ_1-42_/Aβ_1-40_	0.000	0.000–16,876.190	0.391
	p-tau217	2.686	1.003–3.735	**0.014**
	p-tau181	1.002	0.632–1.654	0.181
	GFAP	1.926	1.036–2.384	**0.025**
	NfL	1.017	0.967–1.069	0.970
	PSI3	1.470	1.002–1.978	**0.043**
	PSI2	1.091	0.869–1.345	0.074
	frPSI	1.627	0.979–2.085	**0.035**

A+ MCI, amyloid positive mild cognitive impairment; APOE, Apolipoprotein E; CI, confidence intervals; GFAP, glial fibrillary acidic protein; NfL, Neurofilament Light; OR, odds ratio; PSI, proactive semantic interference; frPSI, failure to recover from proactive semantic interference. Bold values: *p* < 0.05.

### Correlation of semantic intrusion errors and plasma biomarkers across varying amyloid status

**Figure 1:**
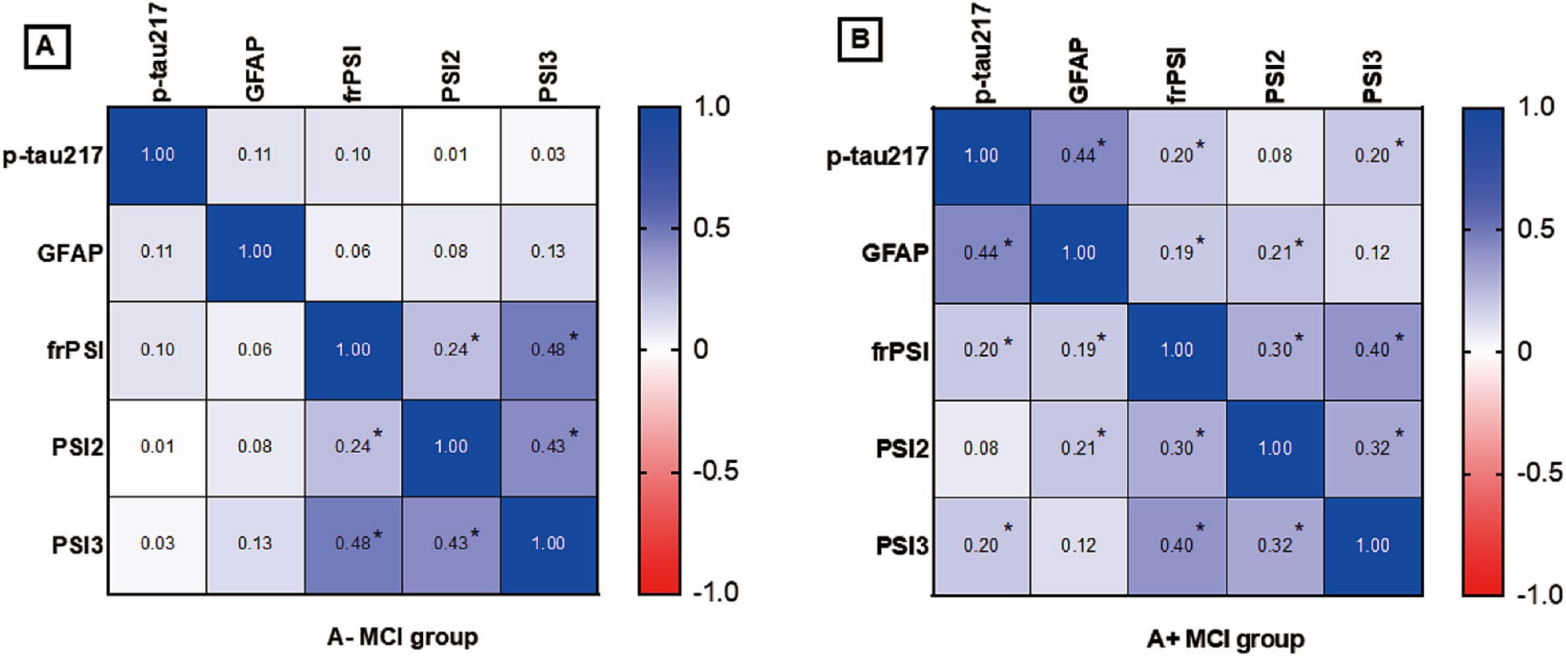
**(A)** Correlation analysis of semantic intrusion errors and plasma biomarkers in A- MCI Group. **(B)** Correlation analysis of semantic intrusion errors and plasma biomarkers in A+ MCI Group. A+ MCI, amyloid positive mild cognitive impairment; A−MCI, amyloid negative mild cognitive impairment; PSI, proactive semantic interference; frPSI, failure to recover from proactive semantic, interference; RSI, retroactive semantic interference; GFAP, glial fibrillary acidic protein. * *p* value< 0.05 by Spearman correlation analysis.

To further explore the extent of co-variation among predictors in the context of amyloid pathology, Spearman correlation analysis was employed for investigation. The heat map of correlation between plasma p-tau217 levels, GFAP levels and SI errors is presented in Figure 1. In the participants who were brain Aβ negative, no significant correlation was observed among plasma p-tau217 levels, GFAP levels, and SI errors. In participants with A+ MCI, p-tau217 levels exhibited significant positive correlations with GFAP levels (*r* = 0.44, *p* = 0.004), frPSI (*r* = 0.200, *p* = 0.009) and PSI3 (*r* = 0.201, *p* = 0.008). In participants with A+ MCI, levels of GFAP demonstrated a significant positive association with both frPSI (correlation coefficient *r* = 0.194, *p* = 0.012) and PSI2 (correlation coefficient *r* = 0.209, *p* = 0.007).

### The combination of semantic intrusion measures and plasma biomarkers in distinguishing between A+ MCI from A− MCI

**Figure 2:**
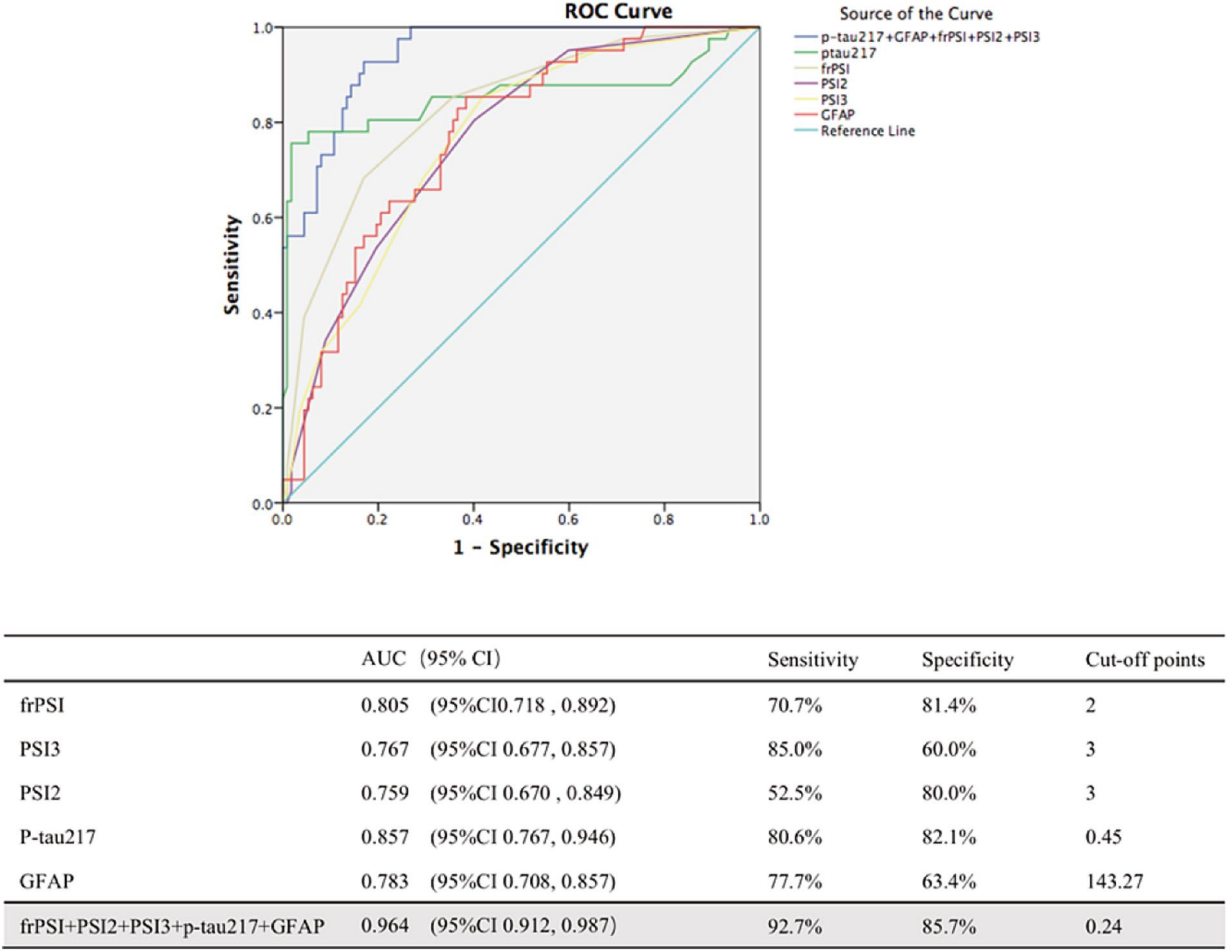
ROC analyses of combination of biomarkers in distinguishing between Amy+ MCI from Amy− MCI. AUC, area under curve; CI, confidence intervals; PSI, proactive semantic interference; frPSI, failure to recover from proactive semantic interference; RSI, retroactive semantic interference; GFAP, glial fibrillary acidic protein.

Among all the types of SI error indicators, PSI2, PSI3 and frPSI show good diagnostic specificity and sensitivity, making BVLT a reliable option for screening amyloid positive MCI patients (Figure 2). For frPSI, ROC analyses for the MCI A+ group vs. the MCI A−group yielded an area under the ROC curve of 0.805 with a binomial exact 95% confidence interval (CI) ranging from 0.718 to 0.892. A cutoff of >2 intrusion errors by yielded a maximum sensitivity of 70.7% and a specificity of 81.4%. P-tau217 and GFAP show good diagnostic specificity and sensitivity in identifying A+ MCI. For p-tau217, ROC analysis for the A+ MCI group vs. the A− MCI group yielded AUC of 0.857 with a binomial exact 95% CI ranging from 0.767 to 0.946. A cutoff of >0.45 yielded a maximum sensitivity of 80.6% and a specificity of 82.1%. The identification of A+ MCI that utilized a combination of multiple predictors demonstrated an improvement in performance over those relying on single predictors. The AUC for identifying A+ MCI using a combination of the PSI2, PSI3, frPSI, P-tau217 and GFAP is 0.964, with a sensitivity of 92.7% and a specificity of 85.7%.

## Discussion

Developing a reliable and cost-effective clinical marker for amyloid positivity could greatly improve the diagnostic process for the prodromal stage of Alzheimer’s disease (AD), particularly for clinicians lacking access to amyloid imaging (Pereira et al., 2018). This study evaluated whether the presence and extent of semantic intrusions on a memory test could identify the amyloid positive MCI using the Bi-list Verbal Learning Test (BVLT). The BVLT, an innovative Chinese adaptation of a cognitive stress test, tailored to the Chinese cultural context, is designed to be challenging while simplifying operational complexity compared to the LASSI-L, thereby ensuring ease of use and minimizing potential errors during the learning process. Detecting semantic interference deficits involves assessing the cued or delayed recall of item intrusions, specifically the number of items from Group B intruded into Group A, or vice versa. Two primary methods for examining semantic interference deficits include measuring correct responses and analyzing semantic interference (SI) errors that occur during recall trials. Previous research has predominantly focused on the number of correct responses elicited by measures of proactive semantic interference (PSI) and retroactive semantic interference (frPSI) (Crocco et al., 2014; Curiel Cid et al., 2019). However, more evidences have suggested that SI errors on PSI and frPSI subscales are better to discriminate between those who are amyloid positive versus amyloid negative MCI (Torres et al., 2019; Kitaigorodsky et al., 2021). In Supplementary Figure 2, individuals with MCI demonstrated poorer performance across almost all the items of the BVLT compared to normal controls. Among the various items in the BVLT, only item N9 demonstrates the ability to distinguish between the A+ MCI and A− MCI groups. When SI errors were examined, there were significant differences in the PSI, frPSI between the A− NC, A+ MCI and A− MCI groups (Table 2). In the prodromal AD stage, patients not only suffer from impaired semantic retrieval but also tend to experience semantic interference (Zhao et al., 2015; Loewenstein et al., 2022; Cui et al., 2024). During the memorization of a large category of words, there is a phenomenon of competitive memory insufficiency among similar vocabulary (Torres et al., 2019).

Delayed recall testing plays a crucial role in the diagnosis of MCI. The operational MCI diagnosing standard takes the score of auditory word delayed recall as the objective evidence of memory deterioration (Guo et al., 2009). A former research has showed that the AVLT long delayed recall (30 min) alone could predict A+ T+ MCI (Stricker et al., 2020). This suggests that delayed recall tests have significant clinical value for the early diagnosis of MCI of AD pathology. In the Table 1, significant differences in AVLT-N3, AVLT-N4, AVLT-N5, AVLT-N7 and MoCA-B were observed between the A− MCI group and the A−NC group, whereas no significance was found between the A− MCI and A+ MCI group. This might be due to the fact that classifying solely based on amyloid positivity diminishes the distinction between groups. The Table 2 shows the comparison of different types of semantic errors and neuropsychology test between A+ MCI, A− MCI, and A− NC. There are significant differences in various types of SI (PSI2, PSI3, frPSI) between the A+MCI and A−MCI group. Notably, these findings were obtained despite no differences in the severity of cognition deficits between the study groups (for example, AVLT, MoCA-B and ACEIII total score). Prior works about the LASSI-L (Matias-Guiu et al., 2018) has consistently shown that frPSI on one additional learning trial is a unique and common feature of MCI due to AD and has been associated with amyloid load (Loewenstein et al., 2018a; Loewenstein et al., 2022; Loewenstein et al., 2016). The BVLT employs the established detection principles of the sematic interference measurements and integrates delayed recall assessments, which enhances the test’s capacity to identify A+ MCI individuals within the Chinese population. Consequently, semantic intrusion errors, as measured not only by frPSI but also by PSI2 and PSI3 in memory tests such as the BVLT, could serve as a cost-effective and readily accessible clinical indicator for predicting amyloid positivity among participants. This has the potential to facilitate screening for therapeutic interventions, making it a valuable tool in the context of clinical trials and routine practice.

The revision of the AA diagnostic criteria in 2024 marks the entry of AD into the era of blood biomarkers. P-tau217 has become a core indicator due to its high sensitivity and ability for early diagnosis. Previous studies have confirmed that in three independent cohorts (*n* = 1,402), plasma p-tau217 distinguished AD from non-AD with an AUC of 0.89–0.96, significantly outperforming p-tau181 [AUC 0.50–0.81 (Palmqvist et al., 2020)]. We used blood biomarkers to differentiate between amyloid-positive (A+) and amyloid-negative (A−) MCI in our study. Thus, the discriminative power of p-tau217 was slightly weaker (AUC = 0.857, with a sensitivity of 80.6%, a specificity of 82.1%). In our study, p-tau217 demonstrated remarkable diagnostic accuracy, surpassing p-tau181, GFAP, and NfL. This aligns with prior findings that plasma p-tau217 excels in detecting the prodromal stage of AD (Palmqvist et al., 2020; Nakamura et al., 2018; Suárez-Calvet et al., 2020; Mattsson-Carlgren et al., 2023). An increasing number of studies have suggested the combination of multiple biomarkers, such as integrating p-tau217, GFAP, Aβ42/40 and various other blood biomarkers, to enhance the specificity of early diagnosis of AD (Janelidze et al., 2023; Milà-Alomà et al., 2022). Figure 1 shows that in the A+ MCI group, the statistically significant positive correlations between p-tau217 and GFAP levels and semantic intrusion errors may indicate that, in the context of amyloid pathology, there is a potential link between these two blood biomarkers and cognitive deficits related to semantic intrusion. Therefore, these indicators may all be sensitive markers for AD-related processes. Compared to participants with A− MCI, those with A+ MCI exhibited stronger positive correlations between p-tau217, GFAP levels and SI errors. This suggests that there is a more specific association within the AD-related MCI subtype. Consequently, by integrating these markers, the study’s most intriguing findings, as depicted in Figure 2, reveal that the synergistic assessment of p-tau217, GFAP levels, and semantic intrusion errors—quantified through PSI2, PSI3, and frPSI—substantially enhances the precision of predicting amyloid status among individuals with Mild Cognitive Impairment (MCI). The AUC for identifying A+ MCI using a combination of multiple biomarkers is 0.964, with a sensitivity of 92.7% and a specificity of 85.7%. A former research has also validated that combining plasma p-tau217, memory tests, executive function assessments and APOE genotyping might greatly improve diagnostic prediction of AD (Palmqvist et al., 2021).

The limitations of the study warrant careful consideration. Firstly, the cross-sectional design implies that the observed differences are more reflective of group characteristics than of disease progression. Consequently, interpretations of the results should be approached with caution, and further validation through larger-scale, longitudinal studies are essential to substantiate the conclusions. Besides, cognitively normal and subjectively cognitive impaired participants with Aβ positivity should be included in future studies to assess whether this pattern can identify early signs of AD pathology even before the onset of overt cognitive impairment. Additionally, while the research included a substantial cohort of well-characterized participants with visual assessments of amyloid burden, it lacked measurements of tau pathology. Furthermore, the study sample predominantly comprised participants from eastern China, highlighting the need for future research that includes a broader range of ethnicities and cultures to validate the generalizability of these findings.

## Conclusion

In conclusion, this study found that, using BVLT measurements, semantic intrusion errors appeared to be an early sign in MCI adults with cerebral amyloid deposition. The combination of p-tau217 levels, GFAP levels and semantic intrusion errors (assessed through PSI2, PSI3 and frPSI) can serve as a robust predictor of amyloid status in individuals with MCI.

## Data Availability

The raw data supporting the conclusions of this article will be made available by the authors, without undue reservation.
