# Supplementary material for: Predicting amyloid status in mild cognitive impairment: the role of semantic intrusions combined with plasma biomarkers
Source: Front Aging Neurosci. 2025 Jun 25;17:1624513. doi: 10.3389/fnagi.2025.1624513 (PMC12237882; doi:10.3389/fnagi.2025.1624513)
Supplement: Supplementary file 1 [file Supplementary_file_1.docx]

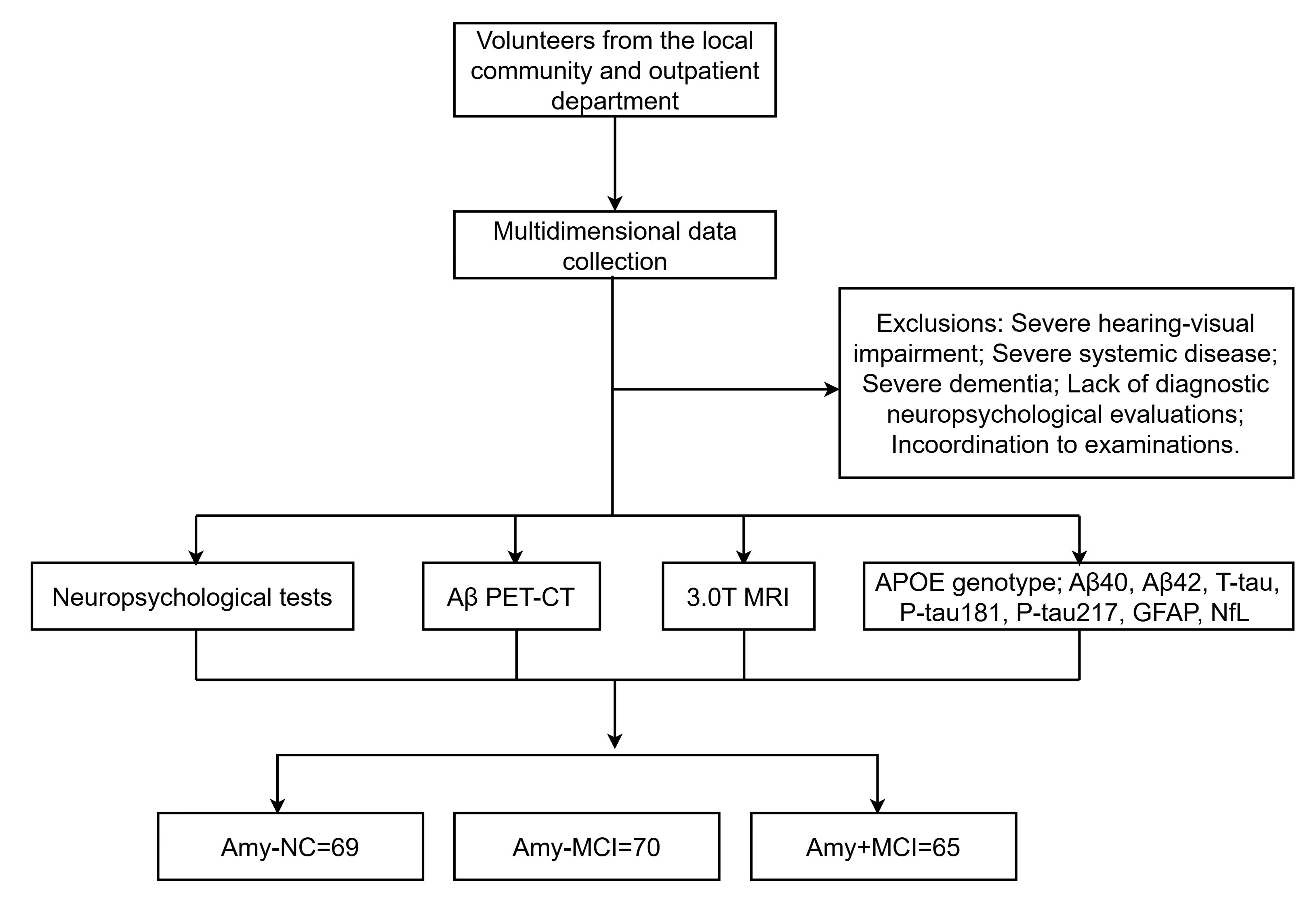


**Supplementary figure 1. Flowcharts of participants.**

Abbreviations: Amy+ = amyloid positive; Amy− = amyloid negative; NC= normal cognition; MCI= mild cognitive impairment; APOE = Apolipoprotein E, NfL = Neurofilament Light, GFAP=glial fibrillary acidic protein
