# Supplementary material for: Predicting amyloid status in mild cognitive impairment: the role of semantic intrusions combined with plasma biomarkers
Source: Front Aging Neurosci. 2025 Jun 25;17:1624513. doi: 10.3389/fnagi.2025.1624513 (PMC12237882; doi:10.3389/fnagi.2025.1624513)
Supplement: Supplementary file 2 [file Supplementary_file_2.docx]

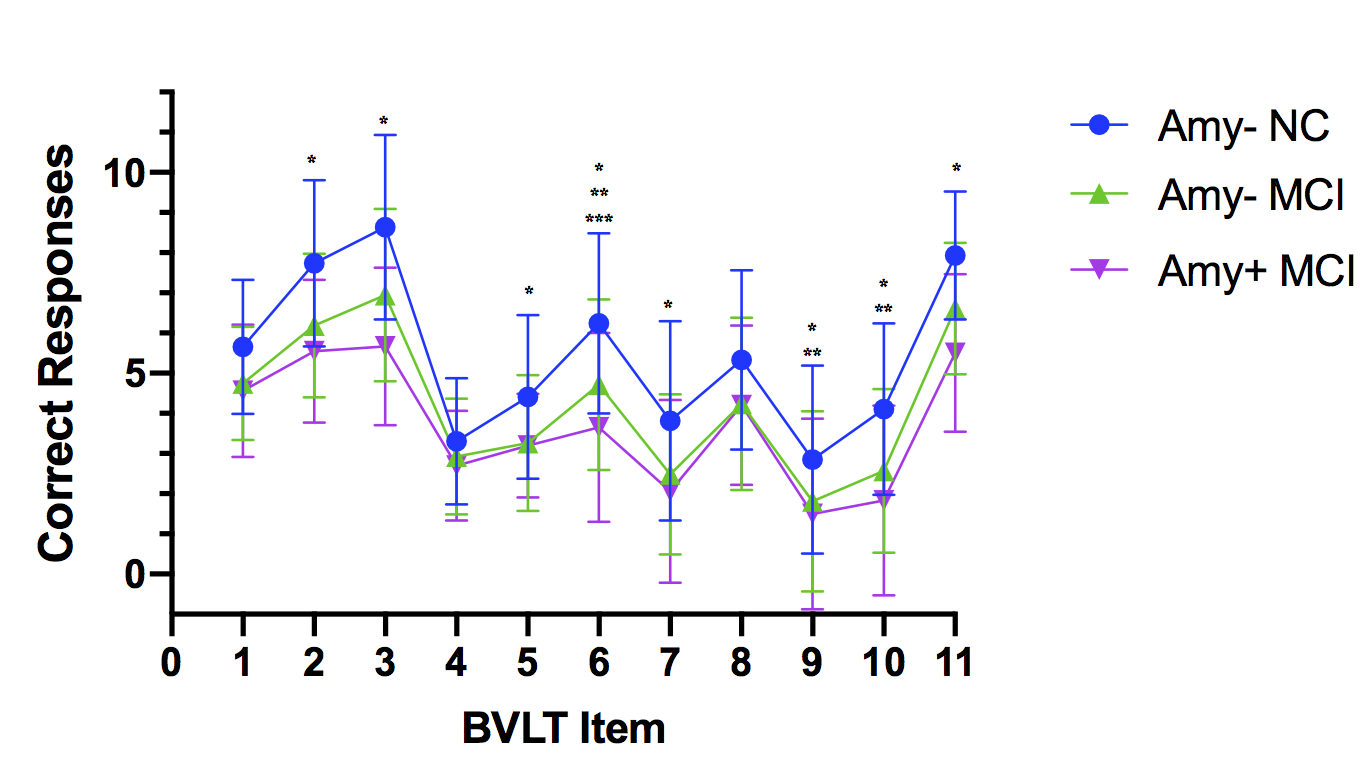


**Supplementary figure 2. Correct responses of each BVLT item of total cohort.**

* : A+ MCI group compared with A- NC group , P<0.05 by the Bonferroni Test

** :A- MCI group compared with A- NC group , P<0.05 by the Bonferroni Test

*** :A- MCI group compared with A+ MCI group , P<0.05 by the Bonferroni Test
